# RBM38 plays a tumor-suppressor role via stabilizing the p53-mdm2 loop function in hepatocellular carcinoma

**DOI:** 10.1186/s13046-018-0852-x

**Published:** 2018-09-03

**Authors:** Jiazhou Ye, Rong Liang, Tao Bai, Yan Lin, Rongyun Mai, Meng Wei, Xinqin Ye, Lequn Li, Feixiang Wu

**Affiliations:** 1 Department of Hepatobiliary Surgery, Affiliated Tumor Hospital of Guangxi Medical University, Guangxi Zhuang Autonomous Region, Nanning, 530021 China; 2 Department of Chemotherapy, Affiliated Tumor Hospital of Guangxi Medical University, Guangxi Zhuang Autonomous Region, Nanning, 530021 China; 3 Department of Pathology, Affiliated Tumor Hospital of Guangxi Medical University, Guangxi Zhuang Autonomous Region, Nanning, 530021 China; 4 Guangxi Liver Cancer Diagnosis and Treatment Engineering and Technology Research Center, Guangxi Zhuang Autonomous Region, Nanning, 530021 China

**Keywords:** RBM38, HCC, P53-mdm2 function loop, Tumor suppressor

## Abstract

**Background:**

Misregulation of the p53-mdm2 loop function is a major mechanism to promote hepatocellular carcinoma (HCC). RBM38, a member of the RNA recognition motif (RRM) family of RNA binding proteins (RBPs), plays a fundamental role in the posttranscriptional control of gene expression and regulatory functions in human tumors. A novel RBM38-p53-mdm2 autoregulatory feedback loop has been demonstrated. However, its mechanistic role in HCC remains unclear.

**Methods:**

In the present study, we investigated the role and molecular mechanism of misregulation in the p53-mdm2 loop function by RBM38 in HCC. First we investigated the correlation of RBM38 activity and p53-mdm2 loop function in liver cancer cells and HCC tissues by western blot and quantitative RT-PCR. We then conducted functional assays to investigate the molecular roles of RBM38 in inhibiting liver cancer cells aggressiveness in vitro and suppressing tumorigenicity in vivo.

**Results:**

We observed RBM38 protein expression was commonly silenced coupled with increased mdm2 and decreased wild type (wt) p53 in liver cancer cells and HCC tissues compared to the corresponding normal liver cells and adjacent liver tissues. RBM38 mRNA level was significantly lower in HCC than adjacent liver tissues, whereas mdm2 and wtp53 mRNA levels were similar between HCC and adjacent liver tissues. This implied that deactivation of RBM38 could disrupt the p53-mdm2 loop and promote HCC, even though p53 and mdm2 transcript amounts were stable. Then, we generated stable liver cancer cell lines with overexpressed RBM38 (RBM38-OE) and found that up-regulation of RBM38 could inhibit mdm2 and restore wtp53 expression. Luciferase assay shown that RBM38 destabilized the mdm2 transcript through binding to multiple AU-/U-rich elements in mdm2 3’-UTR. Furthermore, functional assays showed that ectopic expression of RBM38 could induce liver cancer cell apoptosis and senescence, inhibit proliferation and colony growth, and suppress migration and invasion in vitro. Lastly, RBM38 could suppress HCC tumorigenicity in vivo*.*

**Conclusion:**

Our findings suggested that RBM38 may be a core contributor in stabilizing the p53-mdm2 loop function to prevent HCC, and a potential novel target to provide a therapeutic strategy for HCC by inhibiting mdm2 and rescuing p53 from inactivation.

**Electronic supplementary material:**

The online version of this article (10.1186/s13046-018-0852-x) contains supplementary material, which is available to authorized users.

## Background

Hepatocellular carcinoma (HCC) is the most frequently diagnosed cancer and leading cause of cancer-related deaths worldwide [1]. Despite advances in the diagnosis and treatment of HCC, it remains a major fatal disease. HCC is a heterogeneous disease of complicated etiology due to gene mutations [2], accumulated genetic and epigenetic alterations of various tumor-suppressor genes (TSGs) and oncogenes [3], and dysregulation of coding or non-coding genes [4]. For accurate diagnosis and treatment of HCC, there is an urgent need to precisely understand the molecular mechanisms underlying HCC tumorigenesis and progression.

The wild type (wt) p53 protein acts as a tumor suppressor by initiating cell-cycle arrest apoptosis and senescence in response to cellular stress to maintain the integrity of the genome [5]. It is clear that p53 plays an important role in mitotic fidelity and DNA ploidy conservation in hepatocytes of both normal and regenerative liver. Wtp53 not only restricts malignant transformation, but also promotes a tumor-suppressive microenvironment. Under chronic liver damage, ablation of the p53-dependent apoptosis and senescence program in hepatic stellate cells would enhance the transformation of epithelial liver cells into HCC [6]. Wtp53 is primarily regulated by the E3 ubiquitin ligase Murine Double Minute 2 (MDM2; usually denoted as mdm2 in mice and HDM2 in humans), a transcriptional target and negative regulator of p53. P53 and mdm2 interact with each other to form an autoregulatory feedback loop [7, 8], in which the balance between p53 and mdm2 is critical for regulating cell growth and death in normal cells, and preventing tumors under non-stress conditions or various stimuli [9, 10]. It has been well accepted that misregulation of the p53-mdm2 loop usually leads to mdm2 stabilization and p53 degradation, which plays important and unique roles in tumorigenesis and progression of cancers. Alterations in the mdm2-p53 pathway are common in HCC [11–13], which seems to differ from other types of malignancies for two major reasons. One, 50% of all human tumors carry mutant p53, with frequent p53 mutations occurring in aflatoxin-induced HCC (> 50%) and 20–40% from aflatoxin exposure [14, 15]. Two, mdm2 increased significantly in HCC due to hepatitis virus B and C (HBV and HCV) infections [16, 17], which are the top two risk factors of HCC. When specific hotspots in mdm2 and p53 are exposed to the environmental carcinogen, the p53-mdm2 loop is destabilized and liver cells develop into HCC. Dharel [16] demonstrated that the 309 T > G polymorphism (SNP309, rs2279744), which is located in the intronic p53-responsive promoter of the *mdm2* gene, may increase mdm2 stabilization and accelerate p53 degradation in the early onset of HCC in patients with chronic HCV infection. Yoon [17] evaluated the association of mdm2 and p53 polymorphisms with the early onset of HCC in Korean patients with chronic HBV infection, and found that both the mdm2 SNP309 and the p53 codon 72R > P polymorphism were associated with the development of HCC. Currently, inhibition of mutant p53 remains a hallmark of cancer therapy. The critical role of mdm2-p53 loop in tumor development and progression makes it an exciting target for anticancer drug design. Disruption of the mdm2-p53 interaction by introducing molecules that inhibit mdm2, restore wtp53 and stabilize the active conformation of the p53 protein [14, 18] may offer an effective therapeutic approach, attracting more attention for HCC over recent years [19–21].

Post-transcriptional regulation is emerging as a critical molecular mechanism for gene regulation in mammalian cells [22], has been realized as a novel layer of gene regulation, and is involved in cancer progression [23]. RNA binding proteins (RBPs) play a key role in post-transcriptional control of gene expression, including polyadenylation, RNA splicing, transport, stability, and translation. They contain one or more RNA binding motifs, such as hnRNPK homology motif, RNA recognition motif (RRM), RGG box, and dsRBD motif [22, 24, 25]. RBPs are involved in the expression of various genes responsible for biological processes and cellular functions [22, 24, 25] via deregulation of splicing factors, which might lead to alternative splicing of transcripts and mRNA translation of tumor-suppressor genes or oncogenes in cancer cells [23, 26].The RNA binding motif protein 38 (RBM38) belongs to the RRM family of RBPs, whose gene is located on chromosome 20q13 and expressed in various tissues. RBM38 binding mediates a decrease in mRNA levels and the attenuation of translation [27–29]. In these instances, RBM38 could play pivotal roles in regulating wide biological processes ranging from cell proliferation and cell cycle arrest to cell myogenic differentiation [30, 31]. Recently, Zhang and Xu [32–34] discovered a novel RBM38-mdm2-p53 autoregulatory feedback loop, in which RBM38 is an independent regulator of mdm2 via mRNA stability and p53 via mRNA translation. RBM38 is able to independently inhibit gene and protein expression of mdm2 regardless of p53 by destabilizing its transcript upon binding to multiple AU-/U-rich elements in the three prime untranslated regions (3’-UTR) [32]. Consequently, inhibition of mdm2 may restore p53. In addition, RBM38 can inhibit excessive expression of p53 in a dose-dependent manner by preventing cap-binding protein eIF4E from binding to p53 mRNA [33]. Thus, RBM38 can potentially prevent mdm2 or p53 excessive expression and stabilize the p53-mdm2 loop under non-stress conditions and various stimuli, such as DNA damage, activated mdm2 transcription or p53 accumulation [35]. A study from Ding [36] revealed that RBM38 is inhibited by *HOTAIR* in HCC, and up-regulation of RBM38 could suppress liver cancer cells migration and invasion in vitro. Moreover, Zhang [36] demonstrated that mice deficient in RBM38 are susceptible to spontaneous tumors, which is very similar to mice deficient in p53. Consistent with this, RBM38 may act as a tumor suppressor in HCC by stabilizing the p53-mdm2 loop function. Deactivation of RBM38 could disrupt p53-mdm2 loop function and promote HCC, but its role and molecular mechanism remain scanty and contradictory.

In the present study, we investigated the role and molecular mechanism of RBM38 as a tumor suppressor to prevent HCC via stabilizing the p53-mdm2 loop function. We first investigated the correlation between RBM38 and the onset of HCC. We then generated stable liver cancer cell lines with overexpressed RBM38 and performed functional experiments. Our results demonstrated that RBM38 deactivation could promote HCC tumorigenesis and progression via promoting mdm2, consequently inhibiting p53 and finally disrupting the p53-mdm2 loop function at the posttranscriptional level despite that amounts of p53 and mdm2 transcripts were stable. Functional assays in vitro and in vivo were consistent to show that RBM38 could inhibit mdm2 and rescue p53 functions. Our results strongly suggested that RBM38 is a potential novel target to provide a therapeutic strategy for HCC by inhibiting mdm2 and rescuing p53 from inactivation.

## Methods

### Tissue samples

Snap-frozen HCC and corresponding adjacent normal liver tissues (2-cm distance from tumor) were provided by the Affiliated Tumor Hospital of Guangxi Medical University from July 2016 to December 2016, from patients who underwent liver resection. None of the patients received chemotherapy, radiotherapy or target therapy before liver resection. HCC was confirmed by histopathological examination of hematoxylin-stained paraffin sections, which were individually categorized by independent pathologists. Histologic types were classified according to the World Health Organization (2003). Sample collection was done according to the ethical guidelines of the Declaration of Helsinki and approved by the ethics and research committee of the Affiliated Tumor Hospital of Guangxi Medical University. Before surgery, all of the patients were informed that their surgical specimens would possibly be used for research purposes for inclusion in the data analysis and manuscript publication. Data were analyzed anonymously.

### DNA purification and analysis of exon 7 for p53 mutation

HCC and corresponding adjacent normal liver tissues were collected during liver resection and frozen at − 80 °C. All samples had > 70% viable tumor cells as determined by pathological examination. Genomic DNA was extracted from tumor samples and lymphocytes using the Dneasy Tissue kit (Qiagen, Hilden, Germany) according to the manufacturer’s instructions. Exon 7 of p53 was amplified using primers 5’-CTTGCCACAGGTCTCCCCAA-3′ and 5’-AGGGGTCAGCGGCAAGCAGA-3′, under standard cycling conditions, which yielded a 237-bp PCR product. Purified PCR products were sequenced to evaluate the presence of the Arg → Ser mutation at codon 249.

### Cell lines and cell culture

The human liver cancer cell lines (BEL-7402, BEL-7404, SMMC-7721, MHCC-97H, MHCC-97 L, HepG2, HCCLM3, and Hep-3B) and normal liver cells (L02) were obtained from the American Type Culture Collection (ATCC, VA, USA) and were cultured in High glucose Dulbecco’s Modified Eagle Medium (DMEM) supplemented with 10% fetal bovine serum (FBS) and 1% penicillin-streptomycin solution at 5% CO_2_ and 37 °C.

### Establishment of stable cell lines in which RBM38 or MDM2 was overexpressed

Transfer plasmids and lentivirus packaging cells (Phy-LV029-puro) were provided by Hanyin Co. (Shanghai, China). The RBM38 gene and a negative control sequence (NC) were each cloned into transfer plasmids following the manufacturer’s instructions. Correct clones were verified by sequencing (Hanyin Co., Shanghai, China). To obtain stable cell lines, SMMC-7721 and HepG2 cells were plated in six-well plates and infected with virus and polybrene the following day. Positive clones were selected with puromycin (2 μg/mL and 5 μg/mL for SMMC-7721 and HepG2 cells, respectively) for 14 days to establish the following new stable cell lines: SMMC-7721-RBM38-OE, SMMC-7721-RBM38-NC, HepG2-RBM38-OE, and HepG2-RBM38-NC cells. The efficiency of RBM38 overexpression was confirmed by western blotting.

MDM2 lentivirus was provided by Hanyin Co. (Shanghai, China). SMMC-7721-RBM38-OE and HepG2-RBM38-OE cells were plated in six-well plates and infected with virus and polybrene the following day. Positive clones were selected with puromycin for 14 days to establish the following new stable cell lines: SMMC-7721-RBM38-OE-NC, SMMC-7721-RBM38-OE-MDM2-OE, HepG2-RBM38-OE-NC, and HepG2-RBM38-OE-MDM2-OE cells. The efficiency of MDM2 overexpression was confirmed by RT-PCR and western blotting.

### Generation of reporter vectors

To generate a luciferase reporter carrying mdm2 3’-UTR from nt 1782 to 3433, a DNA fragment was amplified using cDNA samples from SMMC-7721 cells as a template with primers 5’-TTGACCTGTCTATAAGAGAATTATATATTTC-3′ and 5’-GTCTTACGGGTAAATGGTGGCT-3′. The DNA fragment was inserted into pGL3 vector through SpeI and ApaI sites to generate pGL3-MDM2-3UTR-A. The same strategy was done to generate pGL3-MDM2-3UTR-B that contains mdm2 3′-UTR from nt 3412 to 4880, and pGL3-MDM2-3UTR-C that contains the mdm2 3’-UTR from nt 4860 to 5921, using 3’-UTR-B primers 5’-AGCCACCATTTACCCGTAAGAC-3′ and 5’-CAGGCAAACCTTATTCGGCTC-3′, and 3’-UTR-C primers 5’-GAGCCGAATAAGGTTTGCCTG-3′ and 5’-CAGATTCTGCTTGGTTCTAGCTTC-3′.

### RNA extraction, reverse transcription and quantitative RT-PCR (qRT-PCR)

Total RNA was extracted from tissues and cells using Trizol reagent (TaKaRa, A-79061), and cDNA was synthesized using Primescript RT Reagent (TaKaRa) following manufacturer’s instructions. The following PCR primers were used:

- RBM38 forward, 5’-CCACCTTGATCCAGCGGACTTA-3’
- RBM38 reverse, 5’-GCGTGTACTCAATGTAGGGCGA-3’
- P53 forward, 5’-CCTCAGCATCTTATCCGAGTGG-3’
- P53 reverse, 5’-TGGATGGTGGTACAGTCAGAGC-3′
- MDM2 forward, 5’-TGTTTGGCGTGCCAAGCTTCTC-3′
- MDM2 reverse, 5’-CACAGATGTACCTGAGTCCGATG-3′
- β-actin forward, 5’-CACCATTGGCAATGAGCGGTTC-3′
- β-actin reverse, 5’-AGGTCTTTGCGGATGTCCACGT-3′

Quantitative real-time PCR (qRT-PCR) was performed for every cDNA sample on StepOnePlus Real-Time PCR system (Applied Biosystems, USA) using FastStart Universal SYBR Green Master Mix (Roche, Switzerland) according to the manufacturer’s instructions. Quantification was normalized using β-actin as the internal control.

### Western blot analysis

Western blot analysis was performed as described previously [33]. The primary antibodies used were anti-rabbit RBM38 (Abcam), p53 (Santa Cruz) and mdm2 (R&D). The secondary antibodies were purchased from Cell Signaling technology. Band intensities were quantified using densitometric analysis with β-actin (SantaCruz) as the loading control.

### Colony formation assay

Cells were seeded into six-well plates (500 cells/well) and cultured normally for 15–20 days. The colonies were fixed in paraform and stained with Giemsa. The fixed cells were then washed with phosphate-buffered saline (PBS) twice, and dried at room temperature. The colonies in each well were counted, in which all adherent cell colonies contained 50 or more cells.

### Cell counting kit (CCK-8) assay

Cell proliferation was assessed using a CCK-8 kit (Dojindo, Japan) according to the manufacturer’s protocol. Cells were diluted in serum-free medium, and were seeded in a 96-well cell culture plate at 2000 cells/well. The plate was incubated at 37 °C. To measure the growth rate of the cells, 100 μL of spent medium was replaced with an equal volume of fresh medium containing 10% CCK-8; the cells were then incubated further at 37 °C for 3 h. The absorbance was finally determined at 450 nm using a microplate reader (5082Grodig, Tecan, Austria).

### Wound healing assay

SMMC-7721-RBM38-OE, SMMC-7721-NC, HepG2-RBM38-OE, and HepG2-NC cell lines were seeded into six-well plates and were allowed to grow until 100% confluence. The cell layer was then gently scratched through the central axis using a sterile plastic tip and loose cells were washed away. Cell motility was quantified by measuring the distance between the invading fronts of cells in three randomly selected microscopic fields (200×) for each condition and time point (12, 24, 48, and 72 h).

### Cell migration and invasion assays

In vitro cell migration and invasion assays were performed as described previously [32]. Images of three random fields (200×) were captured from each membrane, and the number of migratory or invasive cells was counted.

### β-Galactosidase senescence assays

A total of 1 × 10^6^ SMMC-7721-RBM38-OE, SMMC-7721, HepG2-RBM38-OE and HepG2 cells were each cultured in a 6-cm dish and incubated for 3 days in DMEM supplemented with 10% FBS. When the cells reached approximately 80% confluence, they were fixed and incubated with a freshly prepared senescence-associated β-galactosidase staining solution in the dark at 37 °C overnight. The percentage of cells that were positive for β-galactosidase activity was determined by counting the number of blue cells in 10 fields at 200× magnification.

### DNA histogram analysis

Cell cycle was assessed by flow cytometry (Becton Dickinson, San Jose, CA, USA). For cell cycle analysis, cells were collected, washed with PBS, and fixed in ethanol at − 20 °C for 8 h before being collected by centrifugation. The cells were then washed with PBS, and resuspended in 500 μL of PBS with 0.2% Triton X-100, 10 mM EDTA, 100 μg/mL RNase A, and 50 μg/mL propidium iodide (PI) at room temperature for 30 min.

### Xenograft tumorigenesis in nude mice

BALB/C nude mice (4–6 weeks old, 18–22 g) were randomly divided into two groups (each containing eight mice). Stable SMMC-7721-RBM38-OE and SMMC-7721-NC cells (1 × 10^6^ cells in 0.1 mL PBS) were each injected into the nude mice subcutaneously and tumor growth was followed up for 6 weeks. Tumor volume was measured weekly using a caliper, calculated as (tumor length × width2)/2. Mice were euthanized and checked for final tumor size and volume after six weeks. Mouse studies were conducted according to the Guide for the Care and Use of Laboratory Animals and approved by the Animal Care and Use Committee of Affiliated Tumor Hospital of Guangxi Medical University. Sample collection was done according to the ethical guidelines of the Declaration of Helsinki and approved by the ethics and research committee of the Affiliated Tumor Hospital of Guangxi Medical University.

### Immunohistochemical staining

Hematoxylin & Eosin (H&E) staining was done to observe the histopathology. For immunohistochemical staining, sections were incubated with the primary rabbit antibody of RBM38 (1:100; Abcam) at 4 °C overnight, and subsequently incubated with a biotin-conjugated goat anti-rabbit IgG secondary antibody (1:2500, Promega, USA) for 40–60 min at room temperature before proceeding with the chromogen DAB for the final development.

### TUNEL staining

The sections were mounted on slides to detect apoptosis using the Roche TUNEL staining kit (Penzberg, Germany) according to the manufacturer’s instructions. The TUNEL-positive cells in the cerebral cortices of the penumbra area were counted in five randomized areas per mice tumor, and the results were expressed as the number of stained cells per square meter.

### Statistical analysis

The data were analyzed using the SPSS 17.0 software (SPSS, Chicago, IL, USA). All experiments in this study were repeated in triplicate unless otherwise specified. The Student t-test was used to analyze the statistical significance of the differences between groups. A χ^2^ test and a Fisher Exact test were used to assess the correlation between RBM38 and clinicopathological characteristics. *p* values < 0.05 were considered statistically significant.

## Results

### RBM38 protein was commonly expressed lower in human liver cancer cells and human HCC tissue, coupled with wtp53 inhibition and mdm2 amplification

**Fig. 1:**
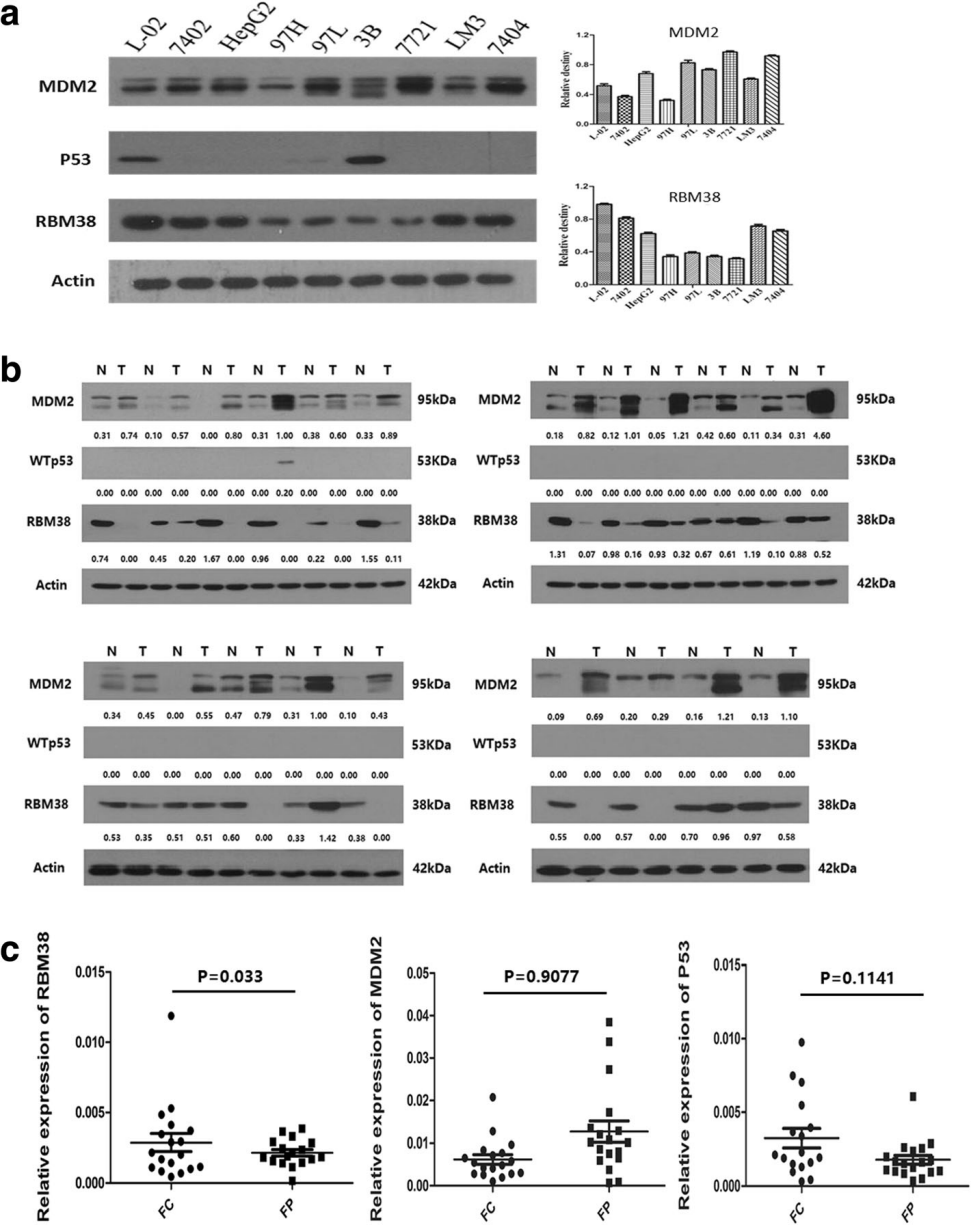
RBM38, wtp53 and mdm2 expression in human HCC and corresponding adjacent liver tissues. **a** Western blot analysis of RBM38, wtp53 and mdm2 expression in human liver cancer cell lines and normal liver cell lines. RBM38 expression was commonly lower in liver cancer cells compared to normal liver cells. Simultaneously, mdm2 protein levels were higher and while wtp53 expression was almost obliterated in all the liver cancer cell lines except Hep-3B. Representative photographs and quantification are shown. **b** Average protein expression levels of RBM38, wtp53 and mdm2 in 24 pairs of HCC and adjacent liver tissue. 21 pairs of HCC and adjacent liver tissue showing decreased RBM38, with increased mdm2 and inhibition of wtp53. Representative photographs and quantification are shown. **c** A scatter plot of RBM38, wtp53 and mdm2 mRNA expression in 24 selected pairs of HCC and adjacent liver tissue. Expression of RBM38 transcripts decreased significantly in HCC tissues (mean: 0.001786 vs. 0.003025, *p =* 0.0033), while both mdm2 or wtp53 mRNA expression in HCC and the corresponding adjacent liver tissues were similar (mdm2: 0.012481 vs. 0.006154, *p =* 0.9077; wtp53: 0.001858 vs. 0.001858, *p =* 0.1141)

First, RBM38, wtp53, and mdm2 protein expression in eight liver cancer cell lines and one normal liver cell line were quantified by western blot (Fig. 1a). Among the nine cell lines analyzed, RBM38 expression was commonly lower in liver cancer cells compared to normal liver cells. Simultaneously, mdm2 protein levels were higher in BEL-7402, SMMC-7721, MHCC-97 L, HepG2, HCCLM3, and Hep-3B liver cancer cell lines compared to the normal liver cell line L-02, while wtp53 expression was almost obliterated in all the liver cancer cell lines except for Hep-3B. Among the liver cancer cells, SMMC7721 cells expressed the highest levels of mdm2.

Next we analyzed exon 7 for p53 mutation in HCC tissues from 62 patients, and detected wtp53 in 29 of the samples. From this subset, wtp53 and mdm2 protein expression were then determined by western blot in HCC tissues and their corresponding adjacent normal liver tissue specimens. Twenty four of the HCC samples contained lower amounts of wtp53 and higher mdm2 protein expression compared to their corresponding adjacent liver tissue. Then, we determined RBM38 protein expression by western blot and IHC staining in these 24 pairs of HCC and corresponding adjacent liver tissue specimens. Western blot showed that RBM38 decreased significantly in 21 HCC tissues compared to their corresponding adjacent liver tissue (Fig. 1b). IHC analysis showed low expression of RBM38 in 21 HCC specimens (+ or ++), while highly expressed in only 3 HCC specimens (+++ or ++++). Representative images are shown in Additional file 1. Finally, we determined RBM38, wtp53 and mdm2 mRNA expression by qRT-PCR in these 24 pairs of HCC and corresponding adjacent liver tissue specimens. The results showed that a significantly lower expression of RBM38 transcripts in the HCC tissues (mean: 0.001786 vs. 0.003025, *p* = 0.0033). Interestingly, both mdm2 or wtp53 mRNA expression in HCC and the corresponding adjacent liver tissues were similar (mdm2: 0.012481 vs. 0.006154, *p* = 0.9077; wtp53: 0.001858 vs. 0.001858, *p* = 0.1141) (Fig. 1c). These results implied that deactivation of RBM38 may promote HCC via disrupting the p53-mdm2 loop function, even though p53 and mdm2 transcript amounts were stable.

### Up-regulation of RBM38 restored wtp53 and inhibited mdm2 expression in human liver cancer cells, but showed no influence on mutant p53 expression

**Fig. 2:**
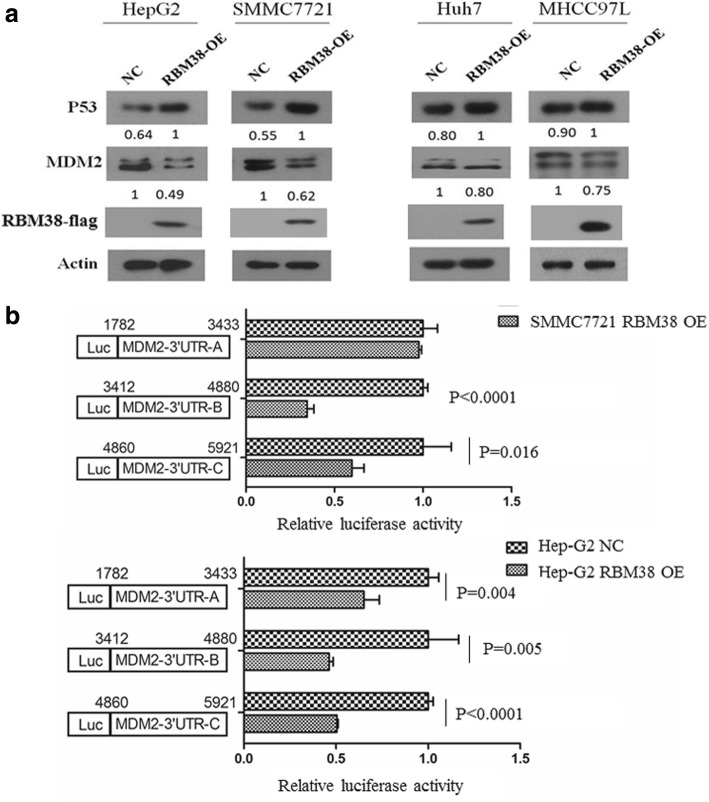
Upregulation of RBM38 increases expression of wtp53 and inhibition expression of mdm2. **a** The fold changes of mdm2, wtp53, RBM38 are shown below each lane. The intensity of the bands were determined using densitometric analysis. Data are representative from three independent experiments. **b** The luciferase activity for the reporter carrying mdm2 3’-UTR-A, -B or -C is repressed by RBM38. SMMC7721-RBM38-OE, SMMC7721-RBM38-NC, HepG2-RBM38-OE, HepG2-RBM38-NC cells were transfected with pGL3 reporter carrying various regions of mdm2 3’-UTR. Cells were then harvested for luciferase assay. The fold change in relative luciferase activity in RBM38-OE cells were compared to NC cells

To address whether the reactivation of RBM38 would restore wtp53 and inhibit mdm2 in liver cancer cells, we infected HepG2 and SMMC7721 cells with lentivirus containing the RBM38 gene and selected for stably infected cells. As illustrated in Fig. 2, up-regulation of RBM38 lead to a significant increase in wtp53 and inhibition of mdm2 protein levels compared to their corresponding controls. Moreover, to determine whether RBM38 has influence on mutant-type p53 (mutp53) expression, we generated Huh-7 and MHCC97-L cell lines that overexpressed RBM38. We found that there was no significant difference in mutp53 expression after up-regulation of RBM38 (Fig. 2a).

Furthermore, to verify that the 3′-UTR in mdm2 transcript is indeed required for RBM38 to inhibit mdm2 expression, a dual-luciferase assay was performed using pGL3 reporters that carry various regions of mdm2 3′-UTR, including 3′-UTR-A, 3′-UTR-B, and 3′-UTR-C, whose sequences are identical to probes A, B, and C, respectively (Fig. 2b). We found that the luciferase activity for a reporter carrying mdm2 3′-UTR-B and -C was significantly repressed by RBM38 in SMMC7721-RBM38-OE cells. By contrast, the mdm2 3′-UTR-A was not responsive to RBM38 (Fig. 2b). In Hep-G2-RBM38-OE cells, the luciferase activities for reporters carrying mdm2 3′-UTR-A, -B and -C were significantly repressed by RBM38 (Fig. 2b).

### Up-regulation of RBM38 inhibited proliferation and growth of human liver cancer cells in vitro

**Fig. 3:**
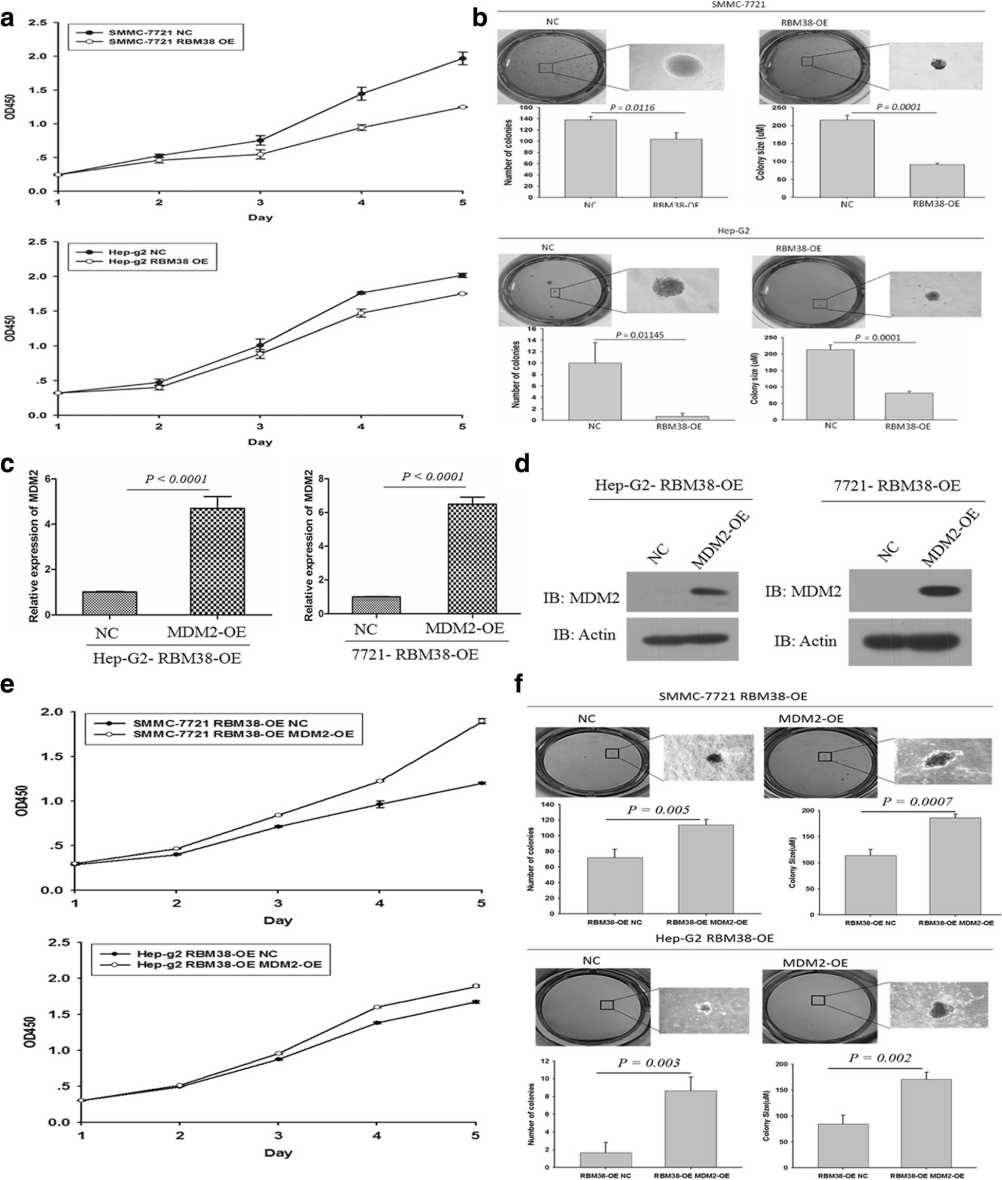
RBM38 suppressed anchorage dependent growth of human liver cancer cells. **a** The growth of cells over 6 days was measured using cell counting kit (CCK-8) assays. RBM38 indicates RBM38 overexpressing SMMC7721 and HepG2 cells; NC indicates SMMC7721 and HepG2 cells transfected with a vector-expressing GFP. The proliferation rate of SMMC7721-RBM38-OE and HepG2-RBM38-OE was significantly decreased compared with control cells, respectively. Data were mean values of three separate experiments mean ± SEM. **b** The growth of cells over 15 days was measured using colony formation assays. Clone formation of RBM38 overexpression arbitrarily set at 100% in control cells (NC). The colony numbers of SMMC7721-RBM38-OE and HepG2-RBM38-OE were significantly reduced and the colony sizes were significantly shrunk compared to control cells, respectively. Representative photographs and quantification are shown. Data were mean values of three separate experiments mean ± SEM. **c**,**d** RT-PCR and Western blotting analysis of mdm2 overexpression in RBM38-OE cells. **e** The growth of cells over 6 days was measured using cell counting kit (CCK-8) assays. The proliferation rates of SMMC7721-RBM38-OE-MDM2-OE and HepG2-RBM38-OE-MDM2-OE were significantly increased compared with control cells, respectively. Data were mean values of three separate experiments mean ± SEM. **f** The growth of cells over 15 days was measured using colony formation assays. The colony numbers of SMMC7721-RBM38-OE-MDM2-OE and HepG2-RBM38-OE-MDM2-OE were significantly rescued and the colony sizes were significantly larger compared to control cells, respectively. Representative photographs and quantification are shown. Data were mean values of three separate experiments mean ± SEM

The growth of the stable cell lines containing over-expressed RBM38 and control cell lines were determined over six days using a Cell counting kit (CCK-8) assay. As shown in Fig. 3a, up-regulation of RBM38 led to significantly decreased cell proliferation in HepG2-RBM38-OE cells (*p =* 0.032) and SMMC7721-RBM38-OE cells (*p =* 0.044) compared to their corresponding control cells. The colony formation assay showed that when RBM38 was over-expressed, the colony number and size were significantly reduced in HepG2-RBM38-OE (colony number, *p =* 0.01145; colony size, *p =* 0.0001) and SMMC7721-RBM38-OE cell lines (colony number, *p =* 0.0116; colony size, *p =* 0.0001) when compared to their corresponding control cells (Fig. 3b).

We overexpressed mdm2 in HepG2-RBM38-OE and SMMC7721-RBM38-OE cells. Both the RT-PCR and western blot results verified the overexpression of mdm2 in these cells compared to control cells (Fig. 3c and d). The growth of the stable cell lines containing mdm2 overexpression in RBM38-OE and control cell lines were determined over six days using a Cell counting kit (CCK-8) assay. As shown in Fig. 3e, high expression of mdm2 regained cell proliferation of HepG2-RBM38-OE cells (*p <* 0.0001) and SMMC7721-RBM38-OE cells (*p =* 0.0002) compared to their corresponding control cells. The colony formation assay showed that when mdm2 was over-expressed in HepG2-RBM38-OE and SMMC7721-RBM38-OE cell lines, the colony number and size were significantly increased in HepG2-RBM38-OE-MDM2-OE (colony number, *p =* 0.005; colony size, *p =* 0.0007) and SMMC7721-RBM38-OE-MDM2-OE cell lines (colony number, *p =* 0.003; colony size, *p =* 0.002) when compared to their corresponding control cells (Fig. 3f).

### Up-regulation of RBM38 induced apoptosis and senescence in human liver cancer cells in vitro

**Fig. 4:**
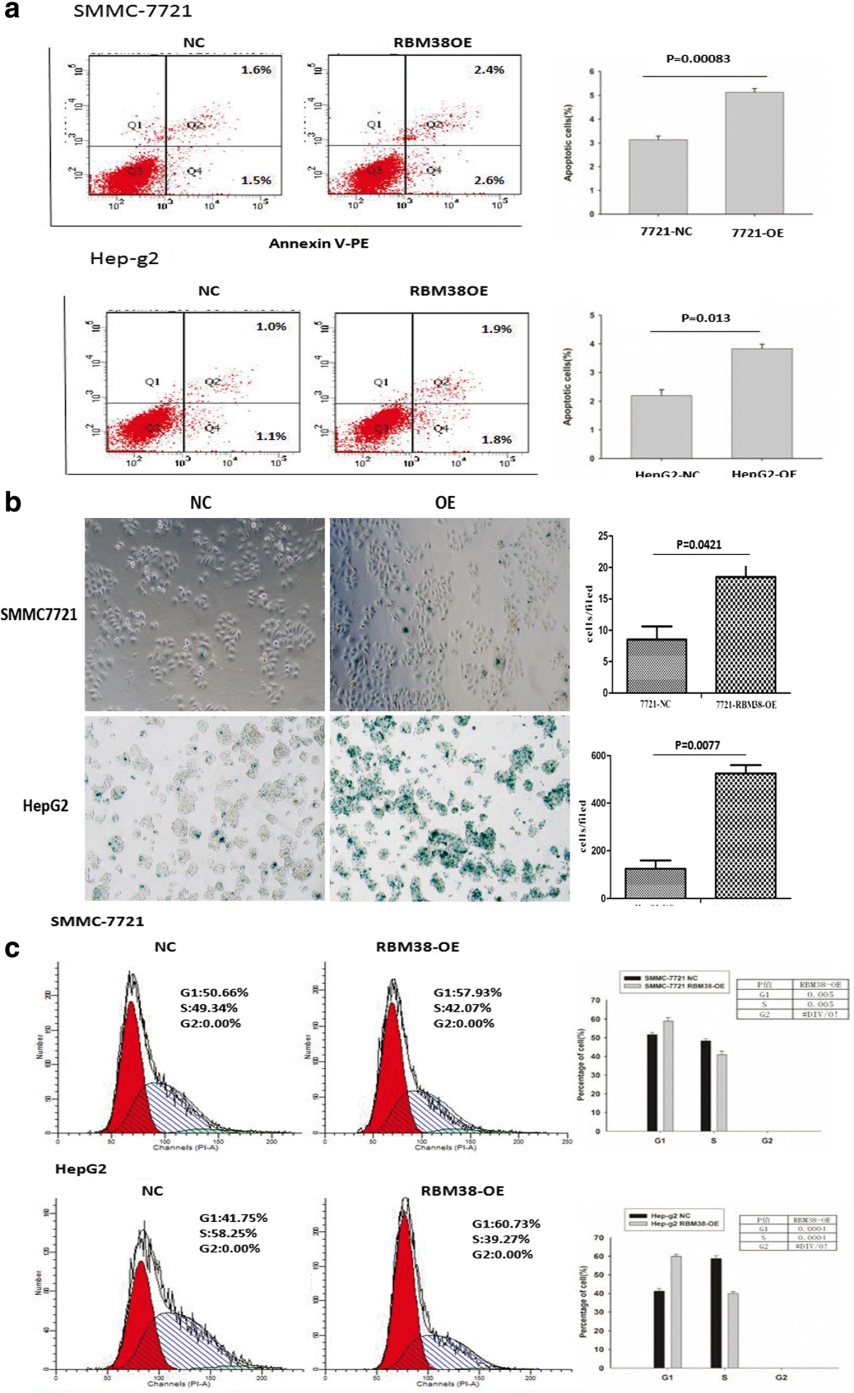
RBM38 induced apoptosis and senescence of human liver cancer cells. **a** Apoptosis was assessed using flow cytometry. The bar graph on the right presents the percentage of apoptotic cells. Representative quadrant figures are presented on the left. The results shown are representative of three independent experiments. **b** Cells were stained with the senescence marker β-galactosidase. The blue staining around the nucleus in SMMC7721-RBM38-OE and HepG2-RBM38-OE cells indicates cellular senescence. **c** Cell cycle progression was measured using flow cytometry. The progression of SMMC7721-RBM38-OE and HepG2-RBM38-OE cells was delayed G1 phase and a shortened S phase compared to control cells, respectively. Representative photographs and quantification are shown

We further evaluated the effect of RBM38 on apoptosis in human cancer cells by flow cytometry. The results demonstrated that up-regulation of RBM38 resulted in the increase of the total number of apoptotic cells in both the HepG2-RBM38-OE (*p =* 0.013) and SMMC7721-RBM38-OE cell lines (*p =* 0.0083) relative to their corresponding control cells (Fig. 4a), indicating that up-regulation of RBM38 could induce apoptosis.

The effect of RBM38 on cell senescence was investigated by β-galactosidase senescence assays. The proportion of cells that were positive for β-galactosidase activity, an indicator of cell senescence, was significantly increased in the HepG2-RBM38-OE (*p =* 0.0077) and SMMC7721-RBM38-OE cell lines (*p =* 0.0421) compared to the corresponding controls (Fig. 4b), suggesting that up-regulation of RBM38 may promote senescence in liver cancer cells, which is consistent with the function of restoring wtp53.

Then, we evaluated the effect of RBM38 on cell cycle progression by using flow cytometry. We found that HepG2-RBM38-OE cells showed a higher number of cells in G1 phase (60.73% vs. 41.75%, *p =* 0.0004) and a lower number of cells in S phase (39.27% vs. 58.25%, *p =* 0.0004) when compared to HepG2-NC cells, while SMMC7721-RBM38-OE cells also showed a higher number of cells in G1 phase (57.93% vs. 50.66%, *p =* 0.005) and a lower number of cells in S phase (42.07% vs. 49.34%, *p =* 0.005) when compared to SMMC7721-NC cells (Fig. 4c). These results suggested that RBM38 inhibited the proliferation and growth, while simultaneously induced the apoptosis and senescence of liver cancer cells potentially via a delay in cell cycle progression.

### Up-regulation of RBM38 suppressed migration and invasion in liver cancer cells

**Fig. 5:**
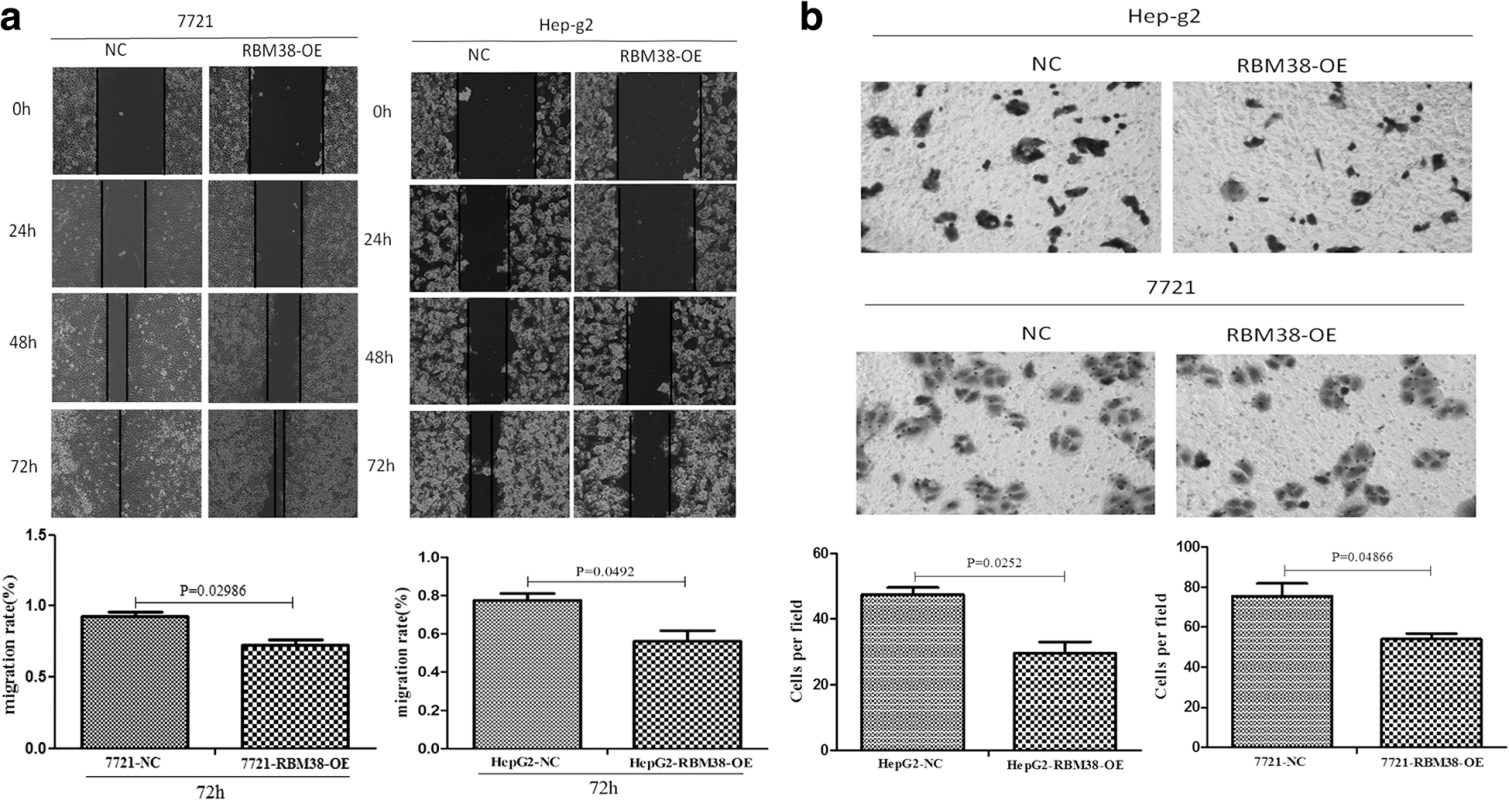
RMB38 significantly decreased migratory and invasive potential of human liver cancer cells. **a** Wound healing assay. Images of wound repair were taken at 0, 24, 48, and 72 h after wound generation. Representative photographs and quantification are shown, original magnification, × 200. **b** Transwell Matrigel invasion assay. Representative photographs and quantification are shown. Columns: average of three independent experiments, original magnification, × 200

We conducted a wound healing assay and a three-dimensional cell invasion assay to examine the effect of RBM38 on migration and invasion in human liver cancer cells. The wound healing assay results showed that the wound gap was markedly larger in the HepG2-RBM38-OE cells (*p =* 0.0298) and SMMC7721-RBM38-OE cells (*p =* 0.0492) compared to their control cells after 72 h (Fig. 5a), indicating that up-regulating RBM38 lowered cell migration rate. In addition, invasion assay results demonstrated that HepG2-RBM38-OE cells (*p =* 0.0252) and SMMC7721-RBM38-OE cells (*p =* 0.0486) exhibited a significant decrease in the ability of invasion compared to their control cells (Fig. 5b).

### RBM38 suppressed tumorigenesis in nude mice

**Fig. 6:**
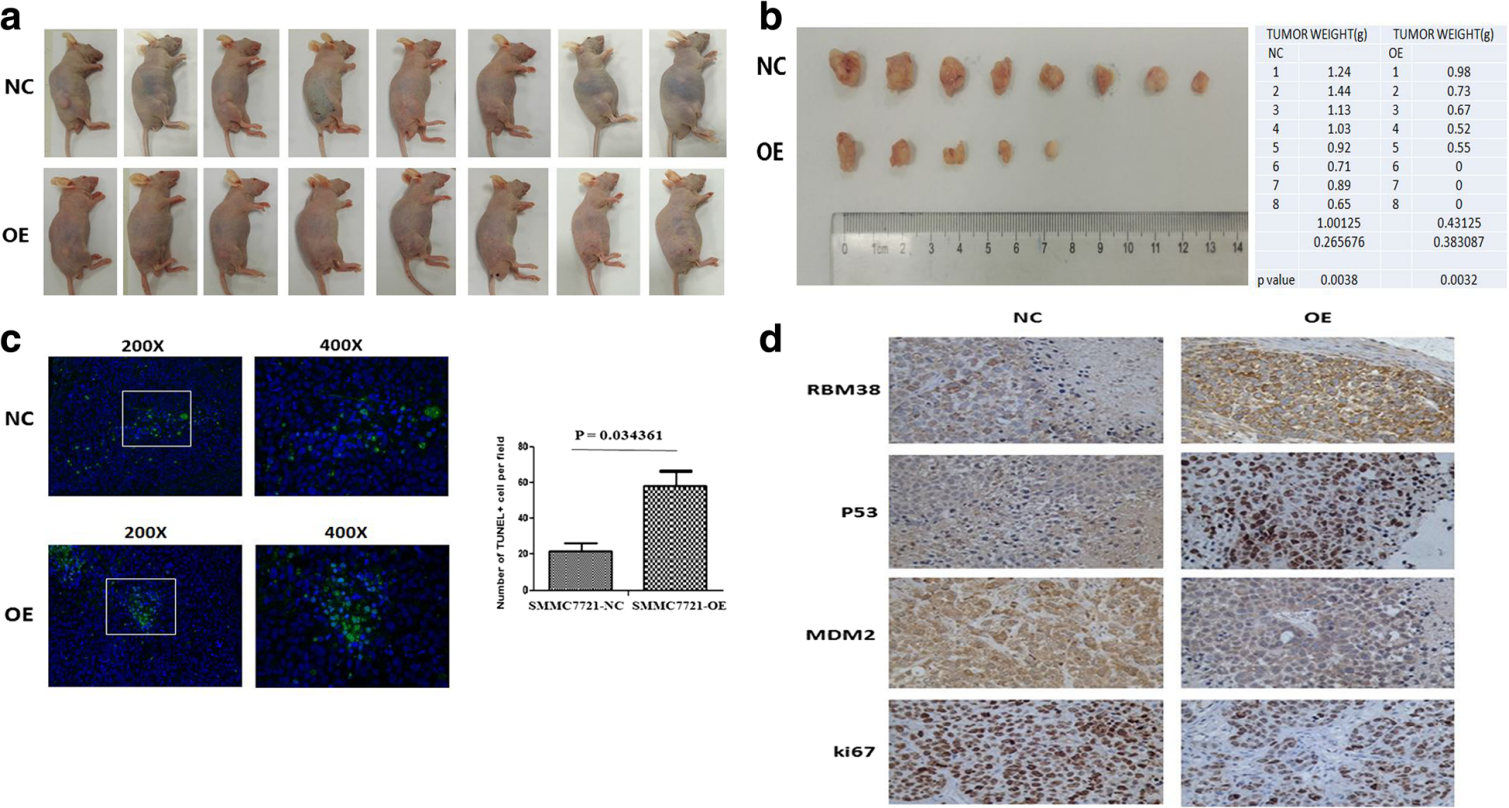
RBM38 suppressed tumor growth in nude mice. **a** The growth of HepG2-RBM38-OE and control cells orthotopically injected into mammary fat pads of the nude mice and the growth of tumors was followed up over 6 weeks. **b** RBM38 over expressed HepG2-RBM38-OE cells formed smaller (*p =* 0.032) and lighter (*p =* 0.038) tumor compared to the control cells (NC). Data were mean values of experiments mean ± SEM. **c** TUNEL (green) in the penumbra area at 72 h post MCAO showing a significant decrease in the number of TUNEL-positive cells in the HepG2-RBM38-OE cells compared to the corresponding controls (*p =* 0.034361). Representative photographs and quantification are shown. Magnification 200×. **d** Immunohistochemical staining showing that tumors originating from HepG2-RBM38-OE cells had increased WTP53 and decreased mdm2 and ki67 levels compared to those originating from control cells, original magnification, × 200

To further evaluate the tumor-suppressive functions of RBM38 in vivo, tumorigenicity of HepG2-RBM38-OE and HepG2-RBM38-NC cells were evaluated in nude mice. Over-expressed RBM38 and control cells were each injected subcutaneously into mammary fat pads of the mice. Tumors derived from over-expressed RBM38 cells were discovered after 4 weeks in five of the mice, while the other mice did not present tumorigenesis. Conversely, tumors derived from the cells of the control group were discovered after 2 weeks (Fig. 6a). Furthermore, tumors from RBM38 over-expressed cells were smaller (*p =* 0.032) and lighter compared to those from the control cells (*p =* 0.038) (Fig. 6b).

In addition, a TUNEL assay was performed at 72 h to determine whether tumorigenesis suppression in nude mice was attributable to apoptosis. As shown in Fig. 6c, the frequency of apoptotic neurons in the penumbra area was decreased significantly in the HepG2-RBM38 cells compared to the corresponding controls, which indicated that tumorigenesis suppression caused by RBM38 up-regulation in nude mice was partly attributable to apoptosis (*p =* 0.03436).

Further, IHC staining revealed that tumor nodules originating from HepG2-RBM38-OE cells had decreased ki67, increased wtp53, and decreased mdm2 expression compared to nodules originating from HepG2-RBM38-NC cells (Fig. 6d). These results were consistent with the in vitro studies, and confirmed that up-regulation of RBM38 decreased tumor growth in vivo in part through re-activation of wtp53 and inhibition of mdm2.

## Discussion

In this study, we investigated the role and molecular mechanism of RBM38 interaction with p53-mdm2 loop in HCC. First, we observed that RBM38 protein expression was commonly decreased in liver cancer cells and HCC compared to normal liver cells and corresponding adjacent liver tissues, while simultaneously associated with increased mdm2 and decreased wtp53. Then, we found that in HCC and corresponding adjacent liver tissues, the mRNA levels of mdm2 and wtp53 were similar, while the RBM38 mRNA level was significantly decreased in HCC compared to those in corresponding adjacent liver tissues. This phenomenon implied deactivation of RBM38 may promote mdm2, then consequently inhibit p53 and finally disrupt the p53-mdm2 loop function at the posttranscriptional level and promote HCC, even though p53 and mdm2 transcript amounts were stable.

Consistent with this, RBM38 may be a potential novel therapeutic target for inhibiting mdm2, rescuing wtp53 from inactivation, and stabilizing the p53-mdm2 loop function. To illustrate this, we generated stable RBM38 over-expressed liver cancer cell lines, and showed that up-regulation of RBM38 could inhibit mdm2 and restore wtp53 expression. We also performed luciferase assays and found that RBM38 destabilizes the mdm2 transcript through binding to multiple AU-/U-rich elements in mdm2 3’-UTR. Furthermore, we conducted functional assays and found that ectopic expression of RBM38 could inhibit liver cancer cell proliferation and growth, induce apoptosis and senescence partly through inducing cell cycle arrest, and suppress migration and invasion in vitro. When mdm2 was up-regulated in RBM38-OE cells, it regained the cell proliferation and growth abilities. Experiments in vivo indicated that RBM38 could suppress tumorigenicity in nude mice. TUNEL staining for the tumors derived from nude mice revealed that this suppression was attributable to apoptosis. Moreover, IHC staining revealed that tumor nodules originating from RBM38 over-expressed liver cancer cells had increased wtp53, decreased mdm2 and ki67 expression compared to nodules originating from the corresponding control cells. Our in vitro and in vivo results were consistent to implied that up-regulation of RBM38 could change the biological functions and progress of HCC in part through inhibition of mdm2 and consequently rescuing wtp53. Similarly, a study from Ding [36] indicated that RBM38 inhibited by *HOTAIR* could induce HCC migration and invasion, and up-regulation of RBM38 could suppress HCC migration and invasion in vitro. However, this study did not show that deactivation of RBM38 could induce mdm2 accumulation and p53 inhibition. Another study from Xue demonstrated that ectopic expression of RBM38 could inhibit breast tumor cell proliferation in vitro partly through inducing cell cycle arrest [18], and could suppress breast tumor cell tumorigenicity in vivo. This study also showed that RBM38 could repress mutp53 in breast cancer, whereas our work showed that RBM38 has no effect on mutp53 in HCC. In addition, a study [33] from Jin and colleagues clearly demonstrated that RBM38 could repress p53 excessive expression by inhibiting p53 mRNA translation. Thus, RBM38 potentially plays a molecular role of regenerating the stability of p53-mdm2 loop, by inhibiting mdm2 and rescuing p53, while preventing excessive activation of p53.

## Conclusion

In summary, our study showed (1) a correlation between RBM38 deactivation and the onset of HCC. Deactivation of RBM38 could promote HCC tumorigenesis and progression via promoting mdm2, then consequently inhibiting p53 and finally disrupting the p53-mdm2 loop function at the posttranscriptional level despite that p53 and mdm2 transcript amounts were stable; (2) increasing RBM38 expression could inhibit mdm2 and restore wtp53 expression in liver cancer cells, as well as (3) suppress their proliferation, growth, migration, invasion, and induce their apoptosis and senescence in vitro; and (4) increasing RBM38 expression suppressed tumorigenicity in vivo. Our results strongly suggest that RBM38 may be a core contributor to stabilizing the p53-mdm2 loop function and a tumor suppressor to prevent HCC. In addition, we propose that RBM38 is a potential novel target for treatment of HCC by inhibiting mdm2 and rescuing p53 from inactivation.

## Additional files

Additional file 1: Images of IHC stained with RBM38 in HCC specimens with scores of + (A), ++(B), +++(C), and ++++(D), original magnification, × 200. (TIF 5956 kb)

## Abbreviations

IHC staining: Immunohistochemical staining
MDM2: Murine Double Minute 2
RBM38: RNA binding motif protein 38
