# Supplementary figures and images for: RBM38 plays a tumor-suppressor role via stabilizing the p53-mdm2 loop function in hepatocellular carcinoma
Source: J Exp Clin Cancer Res. 2018 Sep 3;37:212. doi: 10.1186/s13046-018-0852-x (PMC6122209; doi:10.1186/s13046-018-0852-x)

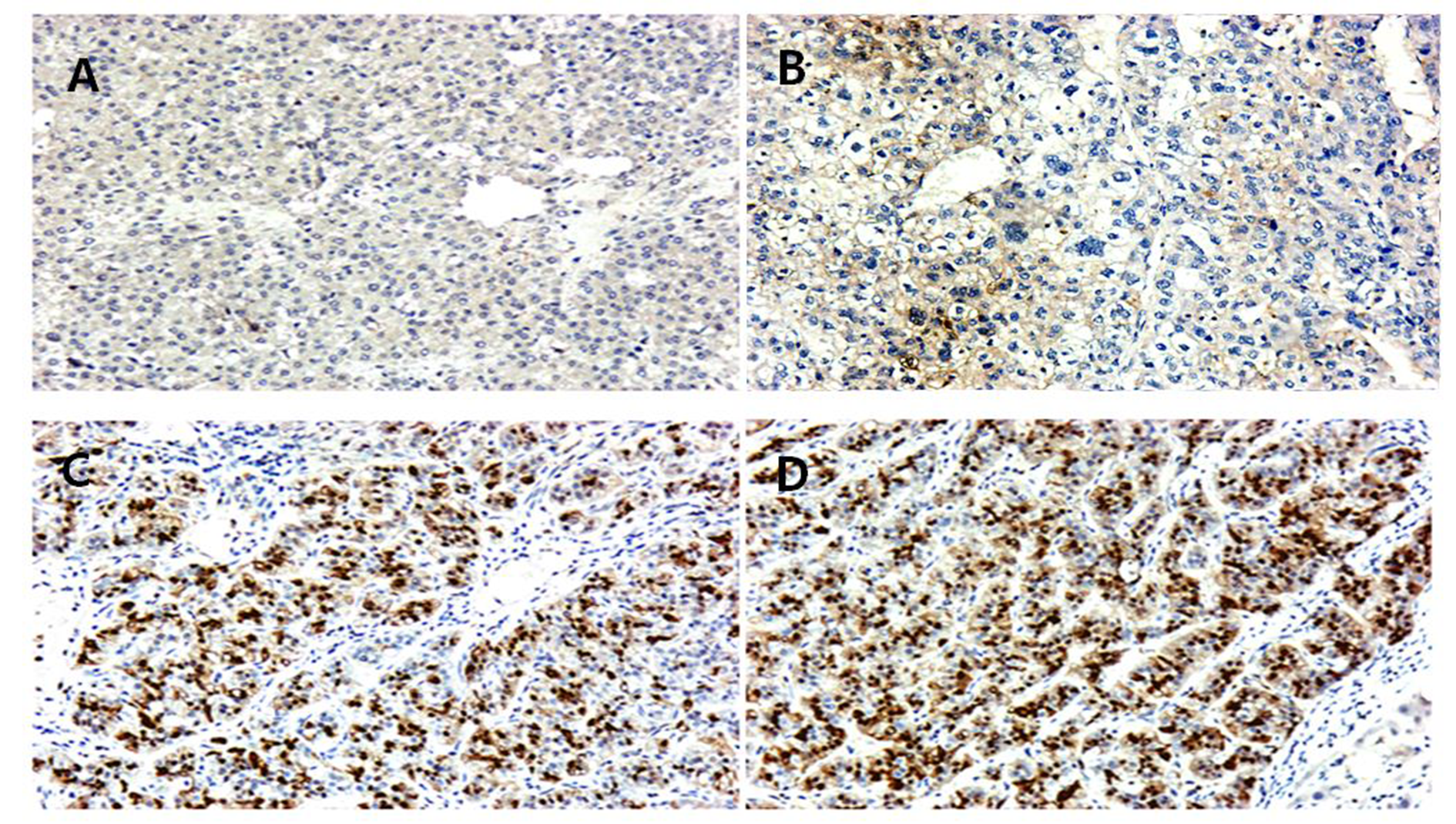

Supplement: Supplementary file 1 — Images of IHC stained with RBM38 in HCC specimens with scores of + (A), ++(B), +++(C), and ++++(D), original magnification, × 200. (TIF 5956 kb) [file 13046_2018_852_MOESM1_ESM.tif]
